# The Effect of Chinese Proficiency on Determining Temporal Adverb Position by Native Japanese Speakers Learning Chinese

**DOI:** 10.3389/fpsyg.2021.783366

**Published:** 2022-01-05

**Authors:** Katsuo Tamaoka, Jingyi Zhang

**Affiliations:** ^1^School of Foreign Languages, Hunan University, Changsha, China; ^2^Graduate School of Humanities, Nagoya University, Nagoya, Japan; ^3^Center for Language and Cultural Studies, University of Miyazaki, Miyazaki, Japan

**Keywords:** temporal adverb, adverb position, Chinese proficiency, native Japanese speakers learning Chinese, Chinese as a foreign language

## Abstract

The present study aimed to investigate how native Japanese speakers learning Chinese choose preferred positions for temporal adverbs depending on their level of Chinese proficiency. A naturalness judgment task conducted on native Chinese speakers showed that the most natural position for Chinese temporal adverbs was before the subject and that placement after the locative prepositional phrase was incorrect. The same task applied to native Japanese speakers found the most natural position for Japanese temporal adverbs was also before the subject. Further, when they appear at the beginning of a sentence, they provide the time for the entire sentence. Accordingly, temporal topicalization appears to influence naturalness decisions by both native Chinese and Japanese speakers. A point of difference was that in Japanese, a temporal adverb placed after a locative prepositional phrase was judged to be acceptable. When the same task was given to native Japanese speakers learning Chinese divided into three Chinese proficiency level groups, placement before the subject was the most preferred by the higher Chinese proficiency group. In addition, placement after the locative prepositional phrase was unfavored by them while the same position was frequently selected by the lower level group. As Chinese proficiency increases it appears that the preferred temporal adverb position is before the subject and the placement after the locative prepositional is judged to be unnatural. Thus, a sense of suitable temporal adverb positions in Chinese is influenced by the level of Chinese proficiency of native Japanese speakers.

## Introduction

Because a verb in a Chinese sentence does not give a clear indication of time, temporal adverbs^1^ play an important role in identifying when an event takes place. Tense in Chinese is typically indicated by temporal adverbs such as *zuótiān* “yesterday” for past tense, *jīntiān* “today” for present tense, and *míngtiān* “tomorrow” for future tense. Textbooks for teaching Chinese as a foreign language (e.g., Liu, 2012; Jiang et al., 2015; Ma et al., 2017; Zhang et al., 2021), related grammar books (e.g., Zhu, 1982; Peng et al., 2005; Qi, 2005; Maruo, 2010; Lu, 2011; Miake, 2012), and linguistic literature (e.g., Zhao, 2001; Liao, 2005; He, 2011; Guan, 2013; Suzuki, 2014; Hirayama, 2017, 2019, Kanemoto, 2018) explain that temporal adverbs are placed either before or after the subject. For instance, “I will drink afternoon tea at the neighbor’s house tomorrow” is expressed in the order of Subject (I) + Adverb (tomorrow) + Prepositional Phrase (at the neighbor’s house) + Verb (drink) + Object (afternoon tea) as in *Wǒ míngtiān zài línjū jiā hē xiàwǔ chá*. The temporal adverb (*Adv*) is placed after the subject “I” and before the locative prepositional phrase (PP) “at the neighbor’s house”, resulting in the order of S*Adv*(time)PP(place)VO. These temporal adverbs may also be placed before the subject in the order of *Adv*(time)S PP(place)VO. However, a temporal adverb occurring after a locative phrase seems to be less preferred (Chao, 2011).

Similarly, temporal adverbs in Japanese are also placed either before or after the subject (Koizumi, 1993; Sun and Koizumi, 2011). For example, a Japanese sentence such as *Ane-ga kinô daigaku-de eigo-no zyugyô-o uke-ta* meaning “Yesterday my older sister took an English class at the university” is ordered S (my older sister) + *Adv* (yesterday) + PP (at the university) + O (an English class) + V (took). The temporal adverb *kinô* “yesterday” may also be placed after the prepositional phrase (PP). Positions of temporal adverbs in Japanese appear to be more flexible than in Chinese.

In contrast, temporal adverbs in English are usually placed at either the beginning or the end of a sentence as in “Yesterday we played golf at the park” or “We played golf at the park yesterday”. Temporal adverbs do not usually appear in the middle of a sentence in English, consequently their placements are not parallel to those of Chinese and Japanese temporal adverbs.

Temporal adverbs in both Chinese and Japanese can be placed in three possible positions, (1) *Adv*(time)S PP(place)VO, (2) S PP(place)*Adv*(time)VO, and (3) S *Adv*(time) PP(place)VO, making Japanese an ideal language for comparison with Chinese. The present study first clarifies differences in temporal adverb positions between Chinese and Japanese. The study then investigates how native Japanese speakers learning Chinese are influenced by their first language. Finally, the study examines how their level of Chinese proficiency influences their choices of preferred positions for temporal adverbs.

## Word Orders in Chinese

Topic-comment structure is often discussed in relation to the Chinese language (e.g., Li and Thompson, 1981; Xu and Langendoen, 1985; Shi, 2000; Chao, 2011). An English question such as ‘Have you finished your homework?’ is asked in Chinese as *Ni zuòwán zuòyè le ma?* S(you) V(finish) O(homework) Asp Q (Asp refers to the aspect *le* while Q refers to the question marker *ma*). However, the object is often made into the topic, especially in conversation, as in *Zuòyè ni zuòwán le ma?* O(homework) S(you) V(finish) Asp Q. In this type of question, the subject *ni* “you” is often dropped when asking the question conversationally. The answer to this question ‘I have finished the homework’ may be in either the canonical SVO order of *Wo zuòwán zuòyè le* S(I) V(finish) O(homework) Asp or the topicalized OSV order of *Zuòyè wo zuòwán le.* O(homework) S(I) V(finish) Asp. Because topicalized structure is frequently seen in Chinese, Chinese is referred to as a topic-comment structure language. Thus, it is hypothesized that, although the canonical word order of Chinese is typically defined as SVO, the topicalized order of OSV, which is frequently observed, is highly accepted.

In relation to Chinese word orders, Sun and Givón (1985) investigated how frequently different word orders occur in Chinese. As Chinese is a pro-drop language (the subject is often omitted), frequencies of VO and OV word orders were counted. The study reported that the VO order was overwhelmingly found in both written and spoken Chinese corpora. More precisely, direct objects occurred after the verb in 94% of expressions in the written corpus and in 92% in the spoken corpus. Conversely, the OV order appeared far less frequently. Sun and Givón (1985) strongly asserted that the canonical order of Chinese is SVO.

Furthermore, a survey of sentence acceptance decisions by Yu and Tamaoka (2018) comparing a single sentence with three different word orders in Chinese (i.e., SVO, SOV, OSV) concluded that SVO was the most acceptable word order in Chinese. Therefore, the unmarked canonical word order of Chinese is identified as SVO while the orders of SOV and OSV are considered to be the marked orders (Chu and Wang, 2016). In sum, topicalization is a marked discourse feature, and non-canonical OSV and SOV orders are based on the distinct information-based structure.

## Topic-Comment Structure Related to Temporal Adverbs

Based on Romance languages such as French, Italian and Spanish, Cinque (1999) proposed a universal hierarchy of functional categories of adverbs. Studies on Japanese adverbs (e.g., Saeki, 1975; Nakau, 1980; Noda, 1984; Kudo, 2000, 2007; Koizumi and Tamaoka, 2006, 2010; Sun and Koizumi, 2011) also indicate that Japanese adverbs fundamentally follow Cingue’s universal hierarchy. Within the framework of this universal hierarchy, Koizumi (1993) asserted that Japanese temporal adverbs are identified as inflectional phrase (IP) adverbs which are positioned either before or after the subject. In fact, an experiment on sentence processing by Koizumi and Tamaoka (2006) indicated that sentences with a temporal adverb before the subject (*tempAdv* + S + O + V: *M* = 1,419 ms, *M* refers to a mean while ms refers to milliseconds) were processed equally as fast as those with a temporal adverb after the subject (S + *tempAdv* + O + V: *M* = 1,401 ms). In turn, both of these were processed significantly faster than those with a temporal adverb after the object (S + O + *tempAdv* + V: *M* = 1,579 ms). Since previous studies on Chinese temporal adverbs (e.g., Zhao, 2001; Liao, 2005; He, 2011; Guan, 2013; Suzuki, 2014; Hirayama, 2017, 2019, Kanemoto, 2018) have proposed that temporal adverbs can be placed either before or after the subject, it seems that temporal adverbs in both Chinese and Japanese can reasonably be classified as IP adverbs.

In accordance with topic-comment structure (Li and Thompson, 1981; Xu and Langendoen, 1985; Shi, 2000; Chao, 2011), native Chinese speakers may prefer to place a temporal adverb before the subject. For example, the action, “my friend eats Shanghai crabs” took place *zuótiān wanshàng* “yesterday night”. In other words, when “yesterday night” is placed at the beginning of the sentence, a listener is able to easily understand that the action of eating Shanghai crabs took place “yesterday night” before listening to the whole sentence. If this is the case, from the production perspective, a speaker will prefer to specify the time first, and then follow that with the content. From the perception perspective, once the time is specified at the beginning of a sentence, a listener can more easily understand the rest of the sentence. This condition fits nicely into the temporal adverb version of topic-comment structure: a temporal adverb becomes the “topic” while the rest of the sentence is a “comment”. In this study, we call this phenomenon “temporal topicalization”. If this assumption of temporal topicalization is true, then native Chinese speakers will prefer the temporal adverb position before the subject rather than after the subject. Furthermore, if the preference for temporal topicalization is a universal phenomenon, native Japanese speakers will also show a preference for placing a temporal adverb before the subject at the beginning of a sentence.

Nevertheless, in order to avoid confusion in word order, temporal adverbs are typically introduced after the subject within the framework of the basic SVO order (e.g., Sun and Givón, 1985; He, 2011; Suzuki, 2014; Hirayama, 2017, 2019; Kanemoto, 2018; Yu and Tamaoka, 2018) in teaching Chinese as a foreign language in a Japanese university. A typical word order is:

(temporal adverb) + Subject + temporal adverb + Verb + Object

(Taken from Kanemoto, 2018, p.30; Suzuki, 2014, p.20)

Locating the adverb before the subject is also possible as indicated by “temporal adverb” in parentheses. Before this point of basic order is taught to native Japanese speakers learning Chinese, their preference may have been to place the temporal adverb after the subject. However, it may be hypothesized that, once Japanese learners encounter more Chinese sentences, they may begin to show a preference for temporal adverbs placed before the subject. As learners’ Chinese proficiency level increased, their choices for sentence naturalness changes gradually from the position after the subject to that before the subject.

Temporal and locative information is also related to word orders. Chao (2011) states that “Other things being equal, there is in Chinese a slight preference for time to come before place” (p. 124). If this is true, then native Chinese speakers should show a lesser degree of preference for temporal adverbs placed after the locative prepositional phrase. Furthermore, Peng et al. (2005) summarized the word order before the verb as follows:

Yǔqì fùcí + Shíjiān + Dìfāng + Duìxiàng Modality + Temporal + Locative + PP(object) + Zěnmeyàng + Dòngcí + Manner/Resultative + Verb

(Taken from Peng et al., 2005, p. 291)

This word order does not include the subject because the subject is often dropped. The subject may be placed either before or after *yuqì fùcí* (modality) or before or after *shíjiān* (temporal). As Chao (2011) suggested, Peng et al. (2005) claimed that in correct word order, *shíjiān* (temporal) comes before *dìfāng* (locative). However, the present study questions whether native Japanese speakers use the same word order for temporal and locative phrases relative to the subject in Japanese, and furthermore, whether those Japanese speakers learning Chinese as a foreign language also show the same tendency in Chinese.

With these assumptions and questions in mind, the present study investigated temporal adverb positions in four steps. In the first step (Experiment 1), temporal adverb positions perceived by native Chinese speakers were tested by a questionnaire survey of naturalness decisions. Experiment 1 clarified the preferred positions of temporal adverbs in Chinese transitive sentences. In the second step (Experiment 2), the same questionnaire survey was given to native Japanese speakers using the equivalent Japanese sentences. In the third step, differences in temporal adverb positions between Chinese and Japanese were compared using these results. In the fourth step, a selection task using three temporal adverb positions of (1) *Adv*(time)S PP(place)VO, (2) S*Adv*(time)PP(place)VO, and (3) S PP(place)*Adv*(time)VO was given to native Japanese speakers learning Chinese. Based on their scores on a Chinese language proficiency test, native Japanese speakers were divided into higher, middle and lower Chinese proficiency groups. Frequencies of selection for temporal adverb positions were then compared among the three groups. Through these steps, the present study clarified how native Japanese speakers learning Chinese were affected by differences in positions of Chinese temporal adverbs according to their level of Chinese proficiency.

## Experiment 1: Preferred Position of Chinese Temporal Adverbs

Using a naturalness decision task, Experiment 1 investigated preferences for Chinese temporal adverb positions by native Chinese speakers.

### Participants

Thirty-eight native Chinese speakers (30 females and 8 males) living in China were recruited online for this study. They participated in Experiment 1 after giving informed consent. They ranged in age from 18 years and 2 months to 58 years and 1 month. The average age was 24 years and 7 months with a standard deviation of 9 years and 6 months on the day the questionnaire was conducted. This study was conducted online, thus enabling participation from all over China. Some participants may have spoken dialects of standard Mandarin Chinese. However, as these dialects are based on common syntactic knowledge, they will not have affected the result of naturalness decisions for written sentences.

### Materials

Stimuli consisted of sentences which included temporal adverbs, in one of three possible positions in a Chinese transitive sentence with a locative prepositional phrase, PP(place) as shown in (1) to (3).


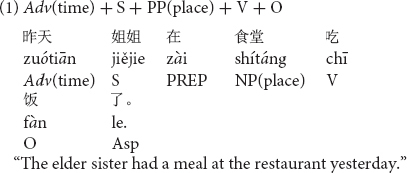



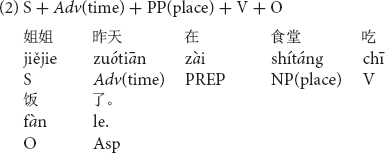



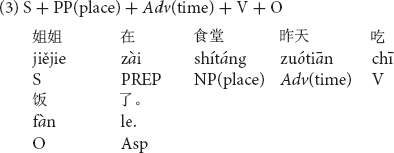


A temporal adverb such as *zuótiān* “yesterday” was placed in the sentence initial position and before the subject in sentence (1), the position after the subject in sentence (2) and the position after a locative prepositional phrase in sentence (3). Using 12 different sentences, each in the three different word orders (36 sentences in total) with temporal adverbs in the past tense were prepared for the first experiment. All stimulus sentences are listed in Supplementary Appendix 1.

### Procedure

Using an online questionnaire survey from (wenjuan.com), all 36 sentences (12 sentences × 3 word orders) were randomly presented to native Chinese speakers for a naturalness decision task. A 5-point Likert scale ranging from −2 (not natural at all) to +2 (completely natural) was used with a positive score indicating a natural range and a negative score indicating an unnatural range. Native Chinese speakers were allowed to respond at their own pace for each question.

### Data Analysis and Results

Means, standard deviations, and standard errors for naturalness judgments by native Chinese speakers for the three temporal adverb positions are reported in Table 1. An analysis of variance with repeated measures was conducted for the three temporal adverb positions. The main effect of the positions was significant in both participant analysis [*F*_1(2,74)_ = 229.32, *p* < 0.001, η_*p*_^2^ = 0.86] and item analysis [*F*_2(2,22)_ = 191.81, *p* < 0.001, η_*p*_^2^ = 0.95].

**TABLE 1:** Naturalness of three temporal adverb positions in Chinese.

Temporal adverb (*adv*) positions	*M*	*SD*	*SE*
*Adv*(time) S PP(place) V O	0.87	0.78	0.13
S *Adv*(time) PP(place) V O	0.68	0.53	0.13
S PP(place) *Adv*(time) V O	–1.40	0.78	0.09

*Adv(time) = temporal adverb. S = subject, PP(place) = locative prepositional phrase, V = verb, and O = object. M = mean, SD = standard deviation, and SE = standard error.*

**FIGURE 1:**
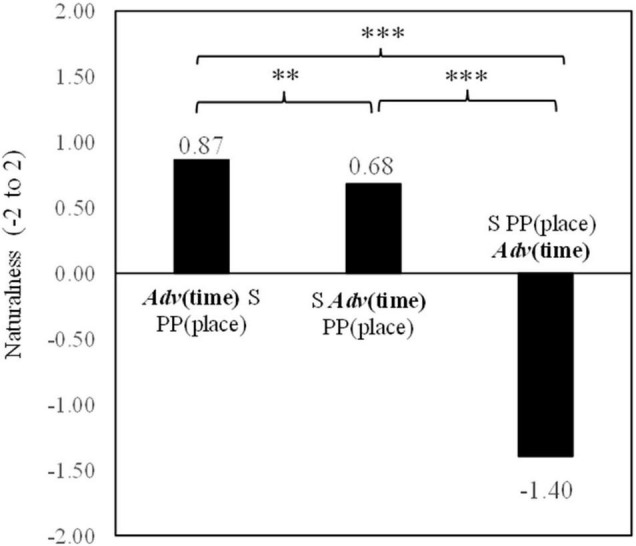
Means of naturalness depending on Chinese temporal adverb positions. **p < 0.01 and ***p < 0.001.

In order to clarify the differences among the three temporal adverb positions, simple contrasts were conducted on the naturalness decisions by native Chinese speakers. As shown in Figure 1 (indicating significances based on the results of simple contrasts in participant analyses), the temporal adverb position before the subject was judged to be more natural than the temporal adverb position after the subject [*F*_1(1,37)_ = 7.84, *p* < 0.01, η_*p*_^2^ = 0.18] but not significant in item analysis [*F*_2(1,11)_ = 2.41, *p* = 0.149, *ns*, η_*p*_^2^ = 0.18]. Item analysis did not reach a significant level. This null significance may have been caused by the small number of sentence stimuli for item analysis. The temporal adverb position before the subject was also judged to be more natural than that after the locative prepositional phrase [*F*_1(1,37)_ = 269.56, *p* < 0.001, η_*p*_^2^ = 0.88; *F*_2(1,11)_ = 281.07, *p* < 0.001, η_*p*_^2^ = 0.96]. Furthermore, the temporal adverb position after the subject was also judged more natural than the temporal adverb position after the locative prepositional phrase [*F*_1(1,37)_ = 243.59, *p* < 0.001, η_*p*_^2^ = 0.87; *F*_2(1,11)_ = 293.21, *p* < 0.001, η_*p*_^2^ = 0.96].

### Discussion

Experiment 1 indicated that the most natural position perceived by native Chinese speakers was the position before the subject. The position after the subject was the second most natural and was also judged as having a high degree of naturalness. This result may be explained by temporal topicalization within the framework of the topic-comment structure in Chinese (Li and Thompson, 1981; Xu and Langendoen, 1985; Shi, 2000; Chao, 2011). The “topic” of a temporal adverb is presented at the beginning of the sentence before the subject, with the rest of the sentence being a “comment”. Native Chinese speakers feel this topic-comment information structure is the most natural. Yet, positions both before and after the subject were still perceived as being in the highly acceptable range. In contrast, the mean of naturalness for the temporal adverb placed after the locative prepositional phrase was rated at −1.40. Since −2.0 indicates an extreme “not natural at all” rating, this score is considered to indicate an incorrect position for placement of a temporal adverb. Against the claim of “a slight preference for time to come before place” by Chao (2011), p. 124, Experiment 1 indicated that native Chinese speakers rejected a temporal adverb placed after a locative prepositional phrase as a constituting a natural sentence.

## Experiment 2: Preferred Position of Japanese Temporal Adverbs

Using a naturalness decision task, Experiment 2 investigated preferences for Japanese temporal adverb positions by native Japanese speakers.

### Participants

One hundred and forty-nine native Japanese speakers (62 females and 87 males) living in Japan and taking a Chinese class at a national university in Japan were recruited online. They participated in Experiment 2 after giving informed consent. They ranged in age from 18 years and 4 months to 36 years and 0 month. The average age was 19 years and 2 months with a standard deviation of 1 years and 6 months on the day the questionnaire was conducted. Most of these native Japanese speakers were from Miyazaki prefecture in Japan. Although accents in this area differ from the Tokyo Standard Japanese, grammatical characteristics are shared by all native Japanese speakers. Therefore, the Japanese participants would not have been affected in judging word orders of temporal adverbs.

### Materials

Stimuli consisted of sentences which included temporal adverbs, in one of three possible positions in a Japanese transitive sentence as shown in (4) to (6).


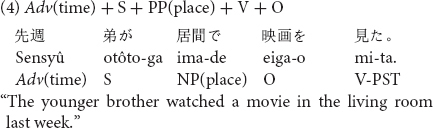



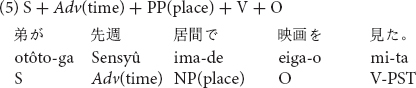



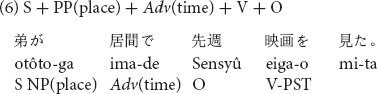


The temporal adverb *sensyû* “last week” was placed before the subject in sentence (4), the position after the subject in sentence (5) and the position after the locative prepositional phrase in sentence (6). Twelve sentences in the three word orders (36 sentences in total) with temporal adverbs of past tense were prepared for Experiment 2. All stimulus sentences are listed in Supplementary Appendix 2.

### Procedure

Using an online questionnaire survey form (Google Forms), 36 sentences (12 sentences × 3 word orders) were randomly presented to native Japanese speakers who were asked to rate how natural a presented sentence was on a 5-point Likert scale from −2 (not natural at all) to + 2 (completely natural). Native Japanese speakers were allowed to respond to each question at their own pace.

### Data Analysis and Results

Means, standard deviations, and standard errors for naturalness judgments by native Japanese speakers for the three temporal adverb positions are reported in Table 2. The analysis of variance with repeated measures was conducted for the three positions in Japanese sentences. The main effect of the positions was significant both in participant analysis [*F*_(2,296)_ = 272.54, *p* < 0.001, η_*p*_^2^ = 0.65] and item analysis [*F*_2(2,22)_ = 472.85, *p* < 0.001, η_*p*_^2^ = 0.98].

**TABLE 2:** Naturalness of three temporal adverb positions in Japanese.

Temporal adverb (*adv*) positions	*M*	*SD*	*SE*
*Adv*(time) S PP(place) V O	1.57	0.52	0.04
S *Adv*(time) PP(place) V O	1.38	0.55	0.05
S PP(place) *Adv*(time) V O	0.08	0.97	0.08

*Adv(time) = temporal adverb. S = subject, PP(place) = locative prepositional phrase, V = verb, and O = object. M = mean, SD = standard deviation, and SE = standard error.*

**FIGURE 2:**
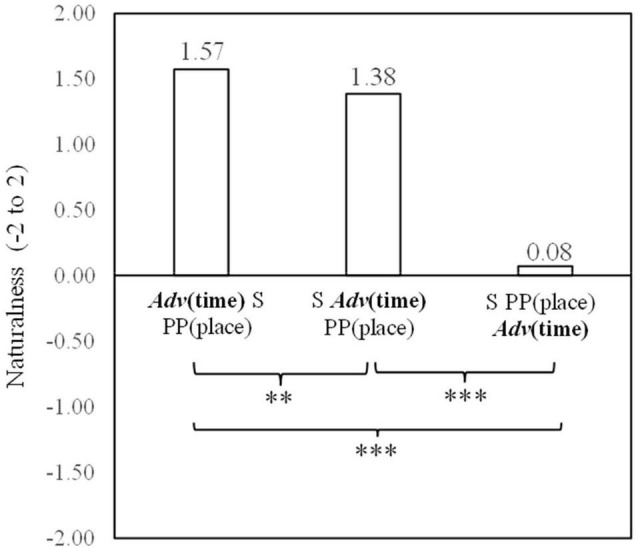
Means of naturalness depending on Japanese temporal adverb positions. **p < 0.01 and ***p < 0.001.

In order to clarify the differences among the three temporal adverb positions, simple contrasts were conducted on the naturalness decisions. As shown in Figure 2, the results showed that the temporal adverb position before the subject was judged to be more natural than that after the subject [*F*_1(1,148)_ = 13.87, *p* < 0.001, η_*p*_^2^ = 0.09; *F*_2(1,11)_ = 10.42, *p* < 0.01, η_*p*_^2^ = 0.49]. The temporal adverb position after the subject was also judged to be more natural than that after the locative prepositional phrase [*F*_1(1,148)_ = 357.41, *p* < 0.001, η_*p*_^2^ = 0.71; *F*_2(1,11)_ = 1157.52, *p* < 0.001, η*_*p*_*^2^ = 0.99]. Furthermore, the temporal adverb position after the subject was judged to be more natural than that after the locative prepositional phrase [*F*_1(1,148)_ = 291.25, *p* < 0.001, η_*p*_^2^ = 0.66; *F*_2(1,11)_ = 535.13, *p* < 0.001, η_*p*_^2^ = 0.98].

### Discussion

Experiment 2 showed that the most natural position perceived by native Japanese speakers was also that of the position before the subject. As with native Chinese speakers, native Japanese speakers also appear to employ temporal topicalization within the framework of a topic-comment structure (Li and Thompson, 1981; Xu and Langendoen, 1985; Shi, 2000; Chao, 2011). A temporal adverb presented at the beginning of a sentence before the subject provides the time when the rest of the sentence takes place. Therefore, temporal topicalization appears to function for naturalness perceived by both native Chinese and native Japanese speakers. The position after the subject was also judged to be the second most natural. This result supports the premise that both Chinese and Japanese temporal adverbs are IP adverbs (Koizumi, 1993). However, unlike in Chinese, the mean of naturalness for the temporal adverb after the locative prepositional phrase had a positive score of 0.08. This rating falls within an acceptable range of naturalness. Therefore, the position after the locative prepositional phrase is not considered to be incorrect in Japanese even though this location is considered to be incorrect in Chinese.

## Naturalness Comparison of Temporal Adverb Positions Between Chinese and Japanese Sentences

The scores for sentence naturalness decisions were compared to confirm the difference in the positions of temporal adverbs between the Chinese and Japanese languages.

### Data Analysis and Results

**FIGURE 3:**
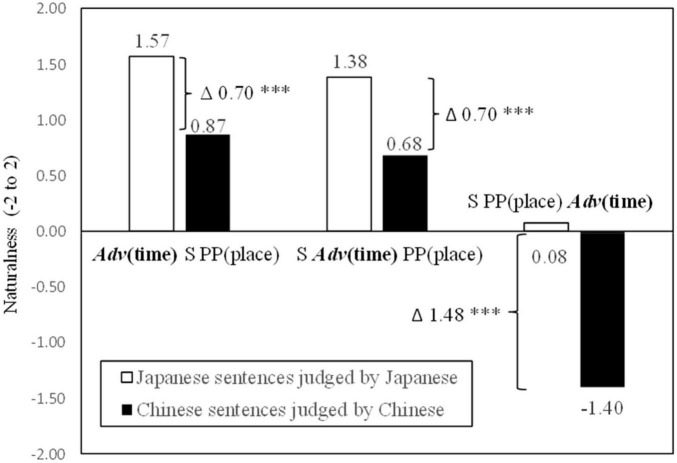
Naturalness scores for Chinese and Japanese temporal adverb positions. Δ indicates the difference in naturalness scores. ^***^ *p* < 0.001.

The mean naturalness scores judged by both native Chinese speakers in Experiment 1 and native Japanese speakers in Experiment 2 for the three temporal adverb positions are depicted in Figure 3. The analysis of variance with repeated measures (the factor of adverb positions was the repeated measure while the factor of first languages was the non-repeated measure) was conducted for the three positions for both Chinese and Japanese sentences. There were significant main effects for positions [*F*_(2,372)_ = 369.35, *p* < 0.001, η_*p*_^2^ = 0.67] and for the participants’ first languages [*F*_(1_,_186)_ = 5574.15, *p* < 0.001, η_*p*_^2^ = 0.97]. Furthermore, there was a significant interaction between the two factors of positions and languages [*F*_(2,372)_ = 18.69, *p* < 0.001, η_*p*_^2^ = 0.09]. These results indicated that (1), as shown in Experiments 1 and 2, temporal adverb positions affect sentence naturalness scores, (2) sentence naturalness scores differ between the Japanese and Chinese languages, and (3) both factors of temporal adverb positions and first languages influence sentence naturalness scores.

After analyzing the differences in the three temporal adverb positions in Experiments 1 and 2, our analysis focused on the differences in naturalness scores between the Chinese and Japanese languages. As shown in Figure 3, the same degree of difference (0.70 points as indicated by Δ in Figure 3) was found in positions both before and after the subject. An independent samples *t*-test was used to analyze the difference in naturalness scores for each temporal adverb position for both native Chinese and Japanese speakers. Due to the fact that Levene’s test for sample distributions indicated differences between samples of native Chinese and Japanese speakers [*F* = 15.93, *p* < 0.001], a *t*-test which does not assume equal distributions was used to analyze scores for the position before the subject. The result indicated a significant difference between the two languages [*t*(47.08) = 6.65, *p* < 0.001, Glass Δ = 0.52]. The effect size was measured by Glass’s delta (Δ), because both sample sizes and sample distributions differed in naturalness scores between native Chinese and Japanese speakers. Likewise, for the position after the subject, Levene’s test for sample distributions indicated differences between the samples of native Chinese and Japanese speakers [*F* = 6.49, *p* < 0.05]. Therefore, a *t*-test which does not assume equal distributions was used. The result indicated a significant difference between the two languages [*t*(48.46) = 3.74, *p* < 0.001, Glass Δ = 0.55].

The largest difference was found in the position after the locative prepositional phrase at 1.48. For this position, once again, Levene’s test for sample distributions indicated differences between samples of native Chinese and Japanese speakers [*F* = 7.75, *p* < 0.01]. Thus, a *t*-test which does not assume equal distributions was applied. The result indicated a significant difference between the two first languages [*t*(84.65) = 10.58, *p* < 0.001, Glass Δ = 0.97].

### Discussion

The difference in naturalness scores for temporal adverb positions before and after the subject was equal at 0.70. This difference may have been a result of decisions made by native Chinese speakers being stricter than those made by native Japanese speakers. However, the difference for the position after the locative prepositional phrase was very large at 1.48. This result suggests that a temporal adverb placed after the locative prepositional phrase in a Chinese sentence is considered incorrect whereas the same order in a Japanese sentence is considered acceptable. A noticeable difference between the Chinese and Japanese languages is seen in the positional relation of a temporal adverb and a locative prepositional phrase.

## Experiment 3: Changes in Temporal Adverb Positions Depending on Chinese Knowledge of Native Japanese Speakers Learning Chinese

Experiment 3 investigated choices of Chinese temporal adverb positions by native Japanese speakers learning Chinese as a foreign language. Based on scores of a Chinese comprehension (or proficiency) test, these choices were analyzed in order to identify the influence of first language based on the results of Experiments 1 and 2, and of their levels of Chinese language proficiency.

### Participants

A total of 149 native Japanese speakers enrolled in a Chinese class as a foreign language at a national university in Japan participated in Experiment 3. They were the same participants who took part in Experiment 2. None of them had been exposed to a Chinese speaking environment. The details of their gender and age are listed in Experiment 2.

### Materials and Procedure

The Chinese stimulus sentences including temporal adverbs were the same as those used in Experiment 1.

### Procedure

Using Google Forms, Japanese participants were asked to choose the most natural sentence in which a temporal adverb was placed in one of three different positions. To ensure they understood the meaning of the words used in the Chinese sentences, the meanings of the words were provided to them. The meanings were presented in the following way: *zuótiān* “yesterday” in Chinese meaning *kinô* in Japanese, *jiìjie* “(my) elder sister” meaning *ane* in Japanese, *shítáng* “restaurant” in Chinese meaning *syokudô* in Japanese, *chī fàn* “to have a meal” in Chinese meaning *gohan-o teberu* in Japanese. A set of three Chinese sentences was then chosen from the sentence stimuli of Experiment 1 (a total of 12 sets shown in Supplementary Appendix 1) and randomly presented to each Japanese participant. Because this study focused on the grammatical aspect of word order, providing lexical meanings would not have affected the results.

### Analysis and Results for Each Set of Sentences

Twelve sets of three sentences containing temporal adverbs were presented to 149 native Japanese speakers resulting in a total of 1,788 responses. Frequencies of sentence choices are reported in Table 3. A Chi-squared test of goodness-of-fit was conducted for each set across the three temporal positions. As shown in Table 3, all sets showed significantly fewer selection frequencies for the position after the locative prepositional phrase. The result showed that Japanese participants consistently chose the temporal adverb location in Chinese, S PP(place) *Adv*(time) O V less frequently in all sentences. The results of a Chi-squared test of goodness-of-fit applied to all 12 sentence sets are shown in Table 3.

**TABLE 3:** Frequencies of selections and the results of Chi-squared test of goodness-of-fit.

	Chinese sentence with temporal adverbs	*Adv*(time) S PP(place)	S *Adv*(time) PP(place)	S PP(place) *Adv*(time)	χ^2^ test of goodness-of-fit
1	姐姐昨天在食堂吃饭了。	59	64	26	χ^2^(2) = 17.17, *p* < 0.001
2	弟弟前天早上在公园玩游戏了。	55	63	31	χ^2^(2) = 11.67, *p* < 0.01
3	妹妹今天早上在教室上汉语课了。	54	70	25	χ^2^(2) = 20.95, *p* < 0.001
4	妈妈昨天上午在超市买牛奶了。	53	69	27	χ^2^(2) = 18.09, *p* < 0.001
5	爸爸昨天晚上在家看电影了。	49	66	34	χ^2^(2) = 10.33, *p* < 0.01
6	哥哥昨天下午在操场踢足球了。	50	70	29	χ^2^(2) = 16.93, *p* < 0.001
7	姐姐今天上午在图书馆写作业了。	50	74	25	χ^2^(2) = 24.17, *p* < 0.001
8	弟弟去年冬天在日本滑雪了。	51	68	30	χ^2^(2) = 14.59, *p* < 0.001
9	奶奶上个星期在饭店吃中国菜了。	56	64	29	χ^2^(2) = 13.54, *p* < 0.001
10	爷爷去年在日本学日语了。	52	70	27	χ^2^(2) = 18.78, *p* < 0.001
11	爸爸前天晚上在公司开会了。	51	65	33	χ^2^(2) = 10.36, *p* < 0.01
12	哥哥前天在学校唱中文歌了。	50	67	32	χ^2^(2) = 12.34, *p* < 0.01
Total		630	810	348	

*The sentences with the temporal adverb position after the subject are presented in the table.*

### Analysis and Results for All Sets Together Based on Chinese Proficiency

To investigate the effect of Chinese proficiency of native Japanese speakers, a Chinese proficiency test was conducted face-to-face in a classroom on the 149 Japanese participants. The test consisted of seven parts: (1) 10 points for lexical knowledge, (2) 10 points for transcription from *pīnyīn* to Chinese characters, (3) 10 points for quantifiers (4) 10 points for sentence types, (5) 10 points for grammatical knowledge, (6) 5 points for Japanese to Chinese translation, and (7) 5 points for understanding of basic conversations. The points in the seven parts added up to a maximum score of 60 points. The Cronbach’s reliability coefficient for the Chinese proficiency test (*N* = 149) was very high at 0.924 (a whole test is provided in Supplementary Appendix 3).

**FIGURE 4:**
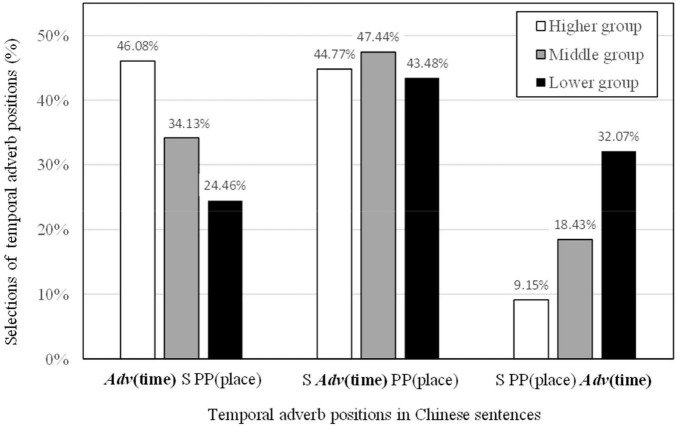
Percentages of frequencies in temporal adverb positions classified by three Chinese proficiency groups.

The mean (*M*) of the Chinese proficiency test for the 149 Japanese participants was 47.75 points with a standard deviation (*SD*) of 9.25 points. Based on the scores of this test, the 149 participants were divided into three groups of higher (*n* = 51, *M* = 56.35, *SD* = 1.87), middle (*n* = 52, *M* = 49.44, *SD* = 2.14), and lower (*n* = 46, *M* = 36.30, *SD* = 7.28) proficiencies. Frequencies of choice for the three temporal positions were calculated for each of the three Chinese proficiency groups. The results of frequencies in percentages are shown in Table 4. The average percentages among the three Chinese proficiency groups across the temporal adverb positions are depicted in Figure 4.

**TABLE 4:** Frequencies and percentages of sentence choices based on Chinese proficiency.

Chinese proficiency	*Adv*(time) S PP(place)		S *Adv*(time) PP(place)		S PP(place) *Adv*(time)		
	*n*	%	*n*	%	*n*	%	Total
Higher group	282	46.08%	274	44.77%	56	9.15%	612
Middle group	213	34.13%	296	47.44%	115	18.43%	624
Lower group	135	24.46%	240	43.48%	177	32.07%	552

The Chi-squared test of independence was conducted on frequencies of choice across the three temporal adverb positions by the three Chinese proficiency groups. The result was significant [χ*^2^*(4) = 118.73, *p* < 0.001]. Because the residuals (*e*) are assumed to be normally distributed, ±1.96 is interpreted to be at the border of the 5% significant level. The standard residuals in Table 5 showed clear tendencies across the three Chinese proficiency groups.

**TABLE 5:** Results of Chi-squared test of independence with standard residuals.

Temporal adverb location		Chinese proficiency			
	Values	Higher group	Middle group	Lower group	Total
*Adv(time*) S PP(place)	Frequency	282	213	135	630
	Expected freq	215.6	219.9	194.5	630
	Std residual	4.5	−0.5	−4.3	
S *Adv(time)* PP(place)	Frequency	274	296	240	810
	Expected freq	277.2	282.7	250.1	810
	Std residual	−0.2	0.8	−0.6	
S PP(place) *Adv(time)*	Frequency	56	115	177	348
	Expected freq	119.1	121.4	107.4	348
	Std residual	−5.8	−0.6	6.7	

*Std residual refers to a standard residual.*

For the position before the subject, the higher level Chinese proficiency group (282 times, *e* = 4.5, *e* refers to the standard residual) selected the temporal adverb before the subject significantly more frequently than did the middle group (213 times, *e* = −0.5). The middle group selected this position even more frequently than did the lower group (135 times, *e* = −4.3). For the position after the subject, all standard residuals were within the range of ±1.96, suggesting no differences in frequencies of choice among the higher (274 times, *e* = −0.2), the middle (296 times, *e* = 0.8) and the lower (240 times, *e* = −0.6) groups. In contrast, for the position after the locative prepositional phrase, the opposite trend was observed. The higher level Chinese proficiency group (56 times, *e* = −5.8) selected the temporal adverb position after the locative prepositional phrase significantly less frequently than did the middle group (115 times, *e* = −0.6). Furthermore, the middle group also selected this position less frequently than did the lower group (177 times, *e* = 6.7).

### Discussion

**FIGURE 5:**
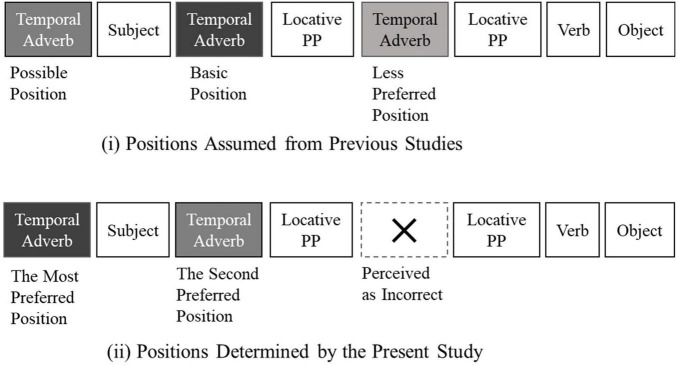
Comparison of temporal adverb positions in Chinese assumed from previous studies and positions identified in Experiment 1.

As shown in the frequency percentages in Figure 5, the three Chinese proficiency groups of native Japanese speakers learning Chinese showed a clear tendency of choice in temporal adverb positions. The higher level Chinese proficiency group chose, in decreasing order of frequencies, the position before the subject, the position after the subject and the position after the locative prepositional phrase. This trend was the same as obtained in Experiment 1 for native Chinese speakers. The higher level group was able to recognize suitable temporal adverb positions at the same rate as demonstrated by native Chinese speakers. The middle Chinese proficiency group most often chose the position after the subject as predicted by previous studies (e.g., Zhao, 2001; Liao, 2005; He, 2011; Guan, 2013; Suzuki, 2014; Hirayama, 2017, 2019; Kanemoto, 2018), but the selection of the position after the locative prepositional phrase was still frequent. This pattern of results suggests some degree of uncertainty in choice of suitable temporal adverb positions. Among the three Chinese proficiency groups, the lower level group most frequently chose the position after the locative prepositional phrase. This location was judged as incorrect by native Chinese speakers in Experiment 1, but perceived as acceptable by native Japanese speakers in Experiment 2. The lower level group may have been influenced by their sense of appropriate temporal adverb position in Japanese.

## General Discussion

The findings of the present study consisted of two parts. The first part relates to temporal adverb positions in Chinese as perceived by native Chinese speakers (Experiment 1) and in Japanese by native Japanese speakers (Experiment 2). The second part relates to choices of Chinese temporal adverb positions by three different Chinese proficiency groups of native Japanese speakers leaning Chinese as a foreign language (Experiment 3). Findings of the two parts are as follows:

### Temporal Adverb Positions in Chinese and Japanese

Based on a naturalness decision task, the present study suggested a clear naturalness preference for Chinese and Japanese temporal adverbs positions.

First, temporal adverb positions both before and after the subject were judged as highly natural by native Chinese speakers. According to the universal hierarchy of functional categories of adverbs (Cinque, 1999), Japanese temporal adverbs are categorized as IP adverbs (Koizumi, 1993; Koizumi and Tamaoka, 2006; Sun and Koizumi, 2011). The results of both Experiment 1 in Chinese and Experiment 2 in Japanese indicated that temporal adverb positions before and after the subject were judged to be highly natural. Therefore, Chinese temporal adverbs appear to also be identifiable as IP adverbs.

Second, the results of both Experiment 1 in Chinese and Experiment 2 in Japanese indicated that sentences with temporal adverbs before the subject were judged to be more natural than those with the adverb following the subject. Previous studies (He, 2011; Suzuki, 2014; Hirayama, 2017, 2019; Kanemoto, 2018) proposed that the basic position of temporal adverbs in Chinese is after the subject although the position before the subject is also acceptable. Other studies (e.g., Zhao, 2001; Liao, 2005; Guan, 2013) have proposed that temporal adverbs can be placed either before or after the subject. However, contrary to assumptions by previous studies depicted in Figure 5(i), the present study, illustrated in Figure 5(ii), concluded that the position before the subject was the most natural. This result may be explained within the framework of the topic-comment structure (Li and Thompson, 1981; Xu and Langendoen, 1985; Shi, 2000; Chao, 2011). A temporal adverb presented at the beginning of the sentence before the subject provides the time at which the rest of the sentence takes place. In this study, this information structure of temporal topicalization strongly appears to influence naturalness decisions by both native Chinese and Japanese speakers.

Third, contrary to the suggestion of “a slight preference for time to come before place” by Chao (2011), a temporal adverb occurring after a locative prepositional phrase was perceived to be very unnatural, or “incorrect”. This phenomenon is illustrated by an X in Figure 5(ii). Since Japanese temporal adverbs occurring after a locative propositional phrase were judged to be within the acceptable range, this unacceptability of this positioning in Chinese appears to be a unique occurrence. Unlike in many languages of the world, a verb in a Chinese sentence does not convey a clear indication of time. Therefore, there seems to be a clear tendency in Chinese for temporal information to be processed before locative information.

In sum, naturalness of temporal adverb positions perceived by native Chinese speakers indicated a clear tendency contrary to the preferred positions assumed from previous studies.

### Preferred Temporal Adverb Positions Based on Chinese Proficiency

Frequency percentages for the three temporal adverb positions selected by native Japanese speakers learning Chinese as a foreign language also displayed a distinct tendency based on their level of Chinese proficiency. There were three major categories of preferred temporal adverb positions: (1) in relation to the locative prepositional phrase, (2) in relation to the position before the subject, and (3) in relation to the position after the subject.

**FIGURE 6:**
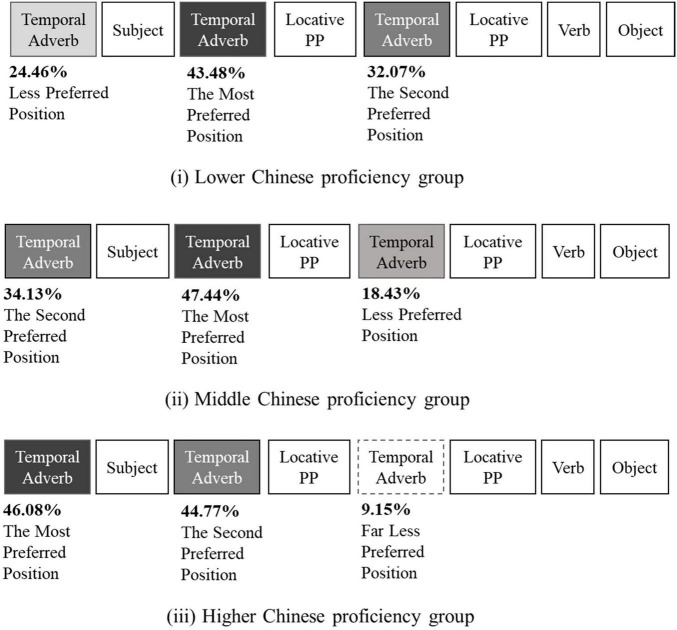
Preferred temporal adverb positions based on levels of Chinese proficiency.

The position after the locative prepositional phrase should be considered to be incorrect as it was perceived to be very unnatural in Experiment 1. However, as illustrated in Figure 6(i), native Japanese speakers with lower Chinese proficiency were most likely to choose this position (32.07%). As Chinese proficiency increased, the percentage selecting this position decreased: 18.43% by native Japanese speakers with the middle Chinese proficiency group in Figure 6(ii) and 9.15% by those in the higher Chinese proficiency group as seen in Figure 6(iii). Because this position was perceived as acceptable in Japanese temporal adverbs in Experiment 2, the lower Chinese proficiency group, and to some degree the middle Chinese proficiency group, may have experienced interference from their first language of Japanese. In contrast, the higher Chinese proficiency group may have acquired a sense that this position is incorrect. Level of proficiency in Chinese clearly reflected the unfavored selection of temporal adverbs positioned after the locative prepositional phrase.

For temporal adverbs occurring before the subject, the higher the level of Chinese proficiency by native Japanese speakers, the more often this was the preferred position. The percentages of selection increased steadily from the lower (24.46%) to the middle (34.13%) to the higher (46.08%) Chinese proficiency level. Those with higher proficiency may have applied temporal topicalization within a topic-comment structure (Li and Thompson, 1981; Xu and Langendoen, 1985; Shi, 2000; Chao, 2011). This information sequence would be applied to temporal adverbs occurring at the beginning of a sentence before the subject. Thus, this appears that a sense of suitable temporal adverb positions in Chinese is influenced by level of Chinese proficiency.

For temporal adverbs positioned after the subject, the order falls within the framework of the SVO basic order. This instructional approach is commonly practiced in order to avoid confusion in word order among Japanese leaners of Chinese as a foreign language in Japanese universities (e.g., Suzuki, 2014; Kanemoto, 2018). As shown in Figure 6, this application seems to be highly accepted by all three Chinese proficiency groups with selection rates at 43.48% by those with lower proficiency, 47.44% by those in the middle group, and 44.77% by those with higher proficiency. The Chinese teaching approach appears to be very effective for native Japanese speakers.

### Implications

Both theoretical and educational implications regarding Chinese word order of temporal adverbs arise from this study.

The theoretical implication of the study is that the topic-comment information structure of topicalization will be viewed as the likely candidate for the determination of word order. In the past, word order of languages has been fundamentally determined based on syntactic structure. However, the present study has suggested that time information is placed at the beginning of the sentence in the form of temporal adverbs. This phenomenon of “temporal topicalization” could be further investigated using both spoken and written corpora. It should be assumed that in spoken language temporal adverbs occur more frequently before the subject while in written language temporal adverbs occur more frequently after the subject. In addition, this use of topicalization most likely reduces the cognitive load for sentence processing. Further studies could enhance our understanding of the efficient production and comprehension of speech, particularly in the context of foreign language acquisition.

An educational implication also arises from the present study. Future educational materials for use by leaners of Chinese as a foreign language could explicitly incorporate the initial findings from this study and from studies subsequent to this one. In particular, the following three points might be included in teaching materials: (1) temporal adverbs can be placed either before or after the subject, (2) temporal adverbs can be placed before the subject in the sentence-initial position as in the English “Last night we ate Shanghai crabs” to clearly indicate the time of an event, and (3) temporal adverbs should not be placed after a locative prepositional phrase.

## Data Availability Statement

The raw data supporting the conclusions of this article will be made available by the authors, without undue reservation.

## Ethics Statement

The studies involving human participants were reviewed and approved by Research Ethics Committee of Humanities Department at Nagoya University. The participants provided their written informed consent to participate in this study.

## Author Contributions

KT constructed the theoretical framework, designed the experiments, performed the statistical analyses, and wrote the draft of the manuscript. JZ conducted the experiments, administered the Chinese comprehension test, and combined the data for the statistical analyses. Both authors contributed to the article and approved the submitted version.

## Conflict of Interest

The authors declare that the research was conducted in the absence of any commercial or financial relationships that could be construed as a potential conflict of interest.

## Publisher’s Note

All claims expressed in this article are solely those of the authors and do not necessarily represent those of their affiliated organizations, or those of the publisher, the editors and the reviewers. Any product that may be evaluated in this article, or claim that may be made by its manufacturer, is not guaranteed or endorsed by the publisher.

## Footnotes

1 Time words such as *zuótiān* “yesterday”, *jīntiān* “today” and *míngtiān* “tomorrow” in Chinese referred to as *shíjiāncí* “time words”. Yuan (2021) explained that *shíjiāncí* is classified as a sub-category of nouns, rather than of adverbs. However, in the present study, we treated time words as temporal adverbs.
