# Supplementary material for: The Effect of Chinese Proficiency on Determining Temporal Adverb Position by Native Japanese Speakers Learning Chinese
Source: Front Psychol. 2022 Jan 5;12:783366. doi: 10.3389/fpsyg.2021.783366 (PMC8769211; doi:10.3389/fpsyg.2021.783366)
Supplement: Supplementary file 1 [file Data_Sheet_1.pdf]

APPENDIX 1: Chinese Stimulus Sentences for Experiment 1

Note: The following 12 sentences are examples of a temporal adverb positioned before the subject.

1. 昨天姐姐在食堂吃饭了。
   Zuótiān jiějie zài shítáng chīfàn le.
   "(My) elder sister had a meal at the restaurant yesterday."
2. 前天早上弟弟在公园玩游戏了。
   Qiántiān zǎoshang dìdi zài gōngyuán wán yóuxì le.
   "(My) younger brother played the game in the park yesterday morning."
3. 今天早上妹妹在教室上汉语课了。
   Jīntiān zǎoshang mèimei zài jiàoshì shàng Hànyǔkè le.
   "(My) younger sister had a Chinese lesson in the classroom this morning."
4. 昨天上午妈妈在超市买牛奶了。
   Zuótiān shàngwǔ māma zài chāoshì mǎi niúnǎi le.
   "(My) mother bought milk at the supermarket yesterday afternoon."
5. 昨天晚上爸爸在家看电影了。
   Zuótiān wǎnshang bàba zài jiā kàn diànyǐng le.
   "(My) father watched a movie at home last night."
6. 昨天下午哥哥在操场踢足球了。
   Zuótiān xiàwǔ gēge zài cāochǎng tī zúqiú le.
   "(My) elder brother played football in the playground yesterday afternoon."
7. 今天上午姐姐在图书馆写作业了。
   Jīntiān shàngwǔ jiějie zài túshūguǎn xiě zuòyè le.
   "(My) elder sister did homework in the library this morning."
8. 去年冬天弟弟在日本滑雪了。
   Qùnián dōngtiān dìdi zài Rìběn huáxuě le.
   "(My) younger brother skied in Japan last winter."
9. 上个星期奶奶在饭店吃中国菜了。
   Shàng ge xīngqī nǎinai zài fàndiàn chī Zhōngguócài le.
   "(My) grandmother ate Chinese food at the restaurant last week."
10. 去年爷爷在日本学日语了。
    Qùnián yéye zài Rìběn xué Rìyǔ le.
    "(My) grandfather studied Japanese in Japan last year."

11. 前天晚上爸爸在公司开会了。

Qiántiān wǎnshang bàba zài gōngsī kāi huì le.

“(My) father had a meeting at the company last night.”

12. 前天哥哥在学校唱中文歌了。

Qiántiān gēge zài xuéxiào chàng Zhōngwéngē le.

“(My) elder brother sang a Chinese song at school the day before yesterday.”
