# Supplementary material for: The Effect of Chinese Proficiency on Determining Temporal Adverb Position by Native Japanese Speakers Learning Chinese
Source: Front Psychol. 2022 Jan 5;12:783366. doi: 10.3389/fpsyg.2021.783366 (PMC8769211; doi:10.3389/fpsyg.2021.783366)
Supplement: Supplementary file 2 [file Data_Sheet_2.pdf]

APPENDIX 2: Japanese Stimulus Sentences for Experiment 2

Note: The following 12 sentences are examples of a temporal adverb positioned before the subject.

1. 先週弟が居間で映画を見た。
   Sensyû otôto-ga ima-de eiga-o mi-ta.
   "(My) younger brother watched a movie in the living room last week."
2. 昨夜妹が台所で髪を乾かした。
   Sakuya imôto-ga daidokoro-de kami-o kawakasi-ta.
   "(My) younger sister dried her hair in the kitchen last night."
3. 先月父が銀行で大金を下ろした。
   Sengetu titi-ga ginkô-de taikin-o orosi-ta.
   "(My) father withdrew a lot of money at the bank last month."
4. 昨日父が公園で財布を落とした。
   Kinô titi-ga kôen-de saihu-o otosi-ta.
   "(My) father dropped his wallet in the park yesterday."
5. 昨夜母がスーパーで牛乳を買った。
   Sakuya haha-ga sûpâ-de gyûnyû-o kat-ta.
   "(My) mother bought milk at the supermarket last night."
6. 去年姉が教会で賛美歌を独唱した。
   Kyonen ane-ga kyôkai-de sanbika-o dokusyôsi-ta.
   "(My) elder sister sang a hymn at the church last year."
7. 昨日母が冷蔵庫でビールを冷やした。
   Kinô haha-ga reizôko-de bîru-o hiyasi-ta.
   "(My) mother chilled the beer in the fridge yesterday."
8. 先週兄がガレージで自転車を直した。
   Sensyû ani-ga garêzi-de zitensya-o naosi-ta.
   "(My) elder brother fixed his bicycle in the garage last week."
9. 去年祖父が北京で中国語を勉強した。
   Kyonen sohu-ga pekin-de tyûgokugo-o benkyôsi-ta.
   "(My) grandfather studied Chinese in Beijing last year."
10. 今朝兄が図書館で宿題を終わらせた。
   Kesa ani-ga tosyokan-de syukudai-o owarase-ta.
   "(My) elder brother finished his homework at the library this morning."
11. 今朝姉がキッチンでコーヒーを入れた。

Kesa ane-ga kittin-de kôhî-o ire-ta.

"(My) elder sister made coffee in the kitchen this morning."

12. 先月祖母がレストランで中華料理を食べた。

    Sengetu sobo-ga resutoran-de tyûkaryôri-o tabe-ta.

    "(My) grandmother ate Chinese food at the restaurant last month."
