# Supplementary material for: The Effect of Chinese Proficiency on Determining Temporal Adverb Position by Native Japanese Speakers Learning Chinese
Source: Front Psychol. 2022 Jan 5;12:783366. doi: 10.3389/fpsyg.2021.783366 (PMC8769211; doi:10.3389/fpsyg.2021.783366)
Supplement: Supplementary file 3 [file Data_Sheet_3.pdf]

Appendix 3: Chinese Proficiency Test in Experiment 3

１. 次の語句を日本語に訳しなさい。（10 点）

1. 上课 ______________________
2. 手机 ______________________
3. 牛奶 ______________________
4. 大家 ______________________
5. 暖和 ______________________
6. 冰箱 ______________________
7. 还可以 ______________________
8. 水果 ______________________
9. 课本 ______________________
10.开空调 ______________________

2. 次のピンインを漢字（簡体字）に直しなさい。（10 点）

1. lǎoshī ______________________
2. dàngāo ______________________
3. yīyuàn ______________________
4. fāyīn ______________________
5. yóupiào ______________________
6. jīntiān ______________________
7. yínháng ______________________
8. xuéxiào ______________________
9. qiānbǐ ______________________
10.diànnǎo ______________________

3. （　　）に適当な量詞を入れなさい （10 点）

1. 房间里有一(　　)床、四(　　)椅子、一(　　)裤子、三(　　)毛衣、一 (　　)西装和一 (　　)狗。

2. 桌子上有两(　　)咖啡、一(　　)牛奶、三(　　)苹果和一(　　)杂志。

4. 下記の単語から最も適当な言葉を選んで（　　　）を埋めなさい（一つの単語の使える回数が限定しません）。（10 点）

姓　　也　　没　　叫　　是不是　　有　　都　　在　　很

1. 食堂(　　)邮局旁边。

2. 汉语语法(　　)难。

3. 她(　　)日本人？

4. 他(　　)铃木，(　　)铃木翼。

5. 她哥哥(　　)她高。

5. **次の文の間違いを直しなさい。**（10 **点**）

1. 超市前边在车站。 ________________

2. 房间里沙发没有。 ________________

3. 面包有冰箱里。 ________________

4. 她们是都中国人。 ________________

5. 日本的冬天冷有点儿。________________

6. 英语不难不？ ________________

7. 你是哪里人吗？ ________________

8. 我家有 5 只人。 ________________

9. 比他的作业我的多。 ________________

10.我有词典一本。 ________________

6. **次の日本語を中国語に訳しなさい。**（5 **点**）

1. これは紅茶ではなく，コーヒーです。

____

2. 私たちの宿題はちょっと多いです。

____

3. スーパーは学校の近くにありますか。（反復疑問文で）

____

4. 冷蔵庫にはパンがありますか。（吗疑問文で）

____

5．彼はチケットを持っていますか。（反復疑問文で）

____

7. **次の問いを中国語で答えなさい。**（5 **点**）

1. 你有几把伞？ ________________

2. 你有没有手机？ ________________

3. 你叫什么名字？ ________________

4. 你家有几口人？ ________________

5. 最近你的作业多吗？ ________________
